# Vestibular Aqueduct Morphology and Meniere's Disease—Development of the “Vestibular Aqueduct Score” by 3D Analysis

**DOI:** 10.3389/fsurg.2022.747517

**Published:** 2022-02-04

**Authors:** Laurent Noyalet, Lukas Ilgen, Miriam Bürklein, Wafaa Shehata-Dieler, Johannes Taeger, Rudolf Hagen, Tilmann Neun, Simon Zabler, Daniel Althoff, Kristen Rak

**Affiliations:** ^1^Department of Oto-Rhino-Laryngology, Plastic, Aesthetic and Reconstructive Head and Neck Surgery and the Comprehensive Hearing Center, University of Würzburg, Würzburg, Germany; ^2^Institute for Diagnostic and Interventional Neuroradiology, University of Würzburg, Würzburg, Germany; ^3^Department of X-ray Microscopy, University of Würzburg, Würzburg, Germany; ^4^Fraunhofer Development Center for X-ray Technology, Würzburg, Germany

**Keywords:** vestibular aqueduct (VA), 3D analysis, temporal bone, saccotomy, computed tomography, Meniere's disease

## Abstract

Improved radiological examinations with newly developed 3D models may increase understanding of Meniere's disease (MD). The morphology and course of the vestibular aqueduct (VA) in the temporal bone might be related to the severity of MD. The presented study explored, if the VA of MD and non-MD patients can be grouped relative to its angle to the semicircular canals (SCC) and length using a 3D model. Scans of temporal bone specimens (TBS) were performed using micro-CT and micro flat panel volume computed tomography (mfpVCT). Furthermore, scans were carried out in patients and TBS by computed tomography (CT). The angle between the VA and the three SCC, as well as the length of the VA were measured. From these data, a 3D model was constructed to develop the vestibular aqueduct score (VAS). Using different imaging modalities it was demonstrated that angle measurements of the VA are reliable and can be effectively used for detailed diagnostic investigation. To test the clinical relevance, the VAS was applied on MD and on non-MD patients. Length and angle values from MD patients differed from non-MD patients. In MD patients, significantly higher numbers of VAs could be assigned to a distinct group of the VAS. In addition, it was tested, whether the outcome of a treatment option for MD can be correlated to the VAS.

## Introduction

Meniere's disease (MD) typically presents as attacks of vertigo lasting from minutes to hours, fluctuating hearing, tinnitus and aural pressure. Variations in these symptoms can make the diagnosis difficult in some cases (1). There are different theories on the pathogenesis of MD based on the endolymphatic hydrops (EH) theory. EH describes an increase of the endolymph inducing an expansion of the cochlear endolymphatic space, resulting in a protrusion of Reissner's membrane into the scala vestibuli.

One hypothesis is, that the EH is triggered by a disturbed absorption of endolymph in the endolymphatic sac (ES). This might happen in patients with a reduced sac lumen, a constricted ductal lumen or a scarred sac.

Another theory states, that the increase of endolymph can also be caused by a disturbance of the active ion transport, which is essential for the composition of the endolymph, resulting in a calcium-rich endolymph and a disturbed electrochemical potential (2). This theory is underlined by the fact, that in patients suffering from MD, the stria vascularis, which regulates the ion concentration, has shown an altered blood supply (3).

In addition, there are different theories attempting to explain how an MD attack develops. An acute attack might occur, when endolymph and perilymph mix due to a permeability disturbance of the inner ear barriers or a rupture of the Reissner's membrane (4). An additional theory postulates, that an MD attack can also occur as a result of blockage of the endolymphatic duct, causing the endolymph to flow in the utricle through the valve of Bast, resulting in vertigo (5).

For diagnosis of MD the AAO-HNS criteria from 1995 (6), later revised in 2015 by the Barany society (7), are commonly used. Based on the duration of vertigo, documented low- to mid-frequency hearing loss and fluctuating aural symptoms like tinnitus or hearing, the patient is categorized in definite or probable MD. Alternatively the Gibson score, which is a 10-point scale of the classical MD symptoms (vertigo, aural fulness, tinnitus and hearing loss) can be used (8).

In addition to the above mentioned clinical symptoms electrocochleography (EcochG) can help in the diagnosis of MD. EcochG refers to the recording of the acoustic evoked cochlear potentials generated by the outer hair cells as well as the auditory nerve action potential. In MD the presence of EH can be evaluated using this technique (9).

3-Tesla-MRI has also proved to be of value in detecting the presence of EH. By local or intravenous application of Gadolinium (Gd), the endolymph can be distracted from the perilymph, since the endolymph has different characteristics of Gd uptake, resulting in an endolymph notch in the Gd rich perilymph (10–12).

Studies have also shown a genetic link to the development of MD. This type of MD, the familial MD, can be linked to different mutated genes or alleles (13).

For MD there are several treatment options, depending on the extent and severity of the symptoms. The current guidelines initially suggest drug therapy. Betahistine in combination with antiemetics and antinauseants for the acute seizure showed good results (14). In case of persistent symptoms, the next step can be a function preserving operation, like the saccotomy, in which the ES is decompressed or opened. This therapy has been reported to be satisfactory, if the ES is not fibrous (15). Another option is transtympanic Gentamycin application, which selectively affects the sensory epithelium of the vestibular organ (16). This mode of therapy results in good vertigo control, with only low risk of consecutively hearing loss (14). When all treatment modalities fail, a neurectomy of the vestibular nerve, or if the patient has already lost hearing, a complete extraction of the membranous labyrinth can be done (17).

The vestibular auquaeduct (VA) is a bony channel that runs from the vestibulum of the inner ear, to the posterior surface of the petrous bone. The VA has an average length of about 6.95–11.86 mm (18, 19) and is divided into proximal short and longer distal parts, which are both positioned at an angle of 90–135° to each other (20). The VA contains the endolymphatic duct (ED), which is filled with endolymph and ends blind as the endolymphatic sac (ES) in a dura duplication. The ES is divided into a proximal, middle (pars rugosa) and distal part. The pars rugosa represents the active part, with a multi-layered secretory epithelium (21). As reported by Eckhard et al. (22), the epithelium of the ES is important for maintaining Na^+^ concentration of the endolymph and volume balance. In addition, this study was the first to describe that the VA in MD patients can be classified into 2 groups; developmentally hypoplastic and degenerative, depending on the angle of the entry and the exit. Particularly noticeable were VA of the hypoplastic type, as these were exclusively found in MD patients with an angle of >140° (20, 22). The authors proposed that due to the reduced area of the epithelium, EH can occur. Similar findings have been previously published describing that the size of the VA influences the extent of the ES (23) and is reduced in MD patients, specifically in volume and length (18, 19). The epithelial layers of the ES in MD have also been described to be pathologically altered. Ikeda and Sando (23) found fibrotic changes with much higher rates in the VA of MD patients.

Based on these studies, the aim of the present paper was to investigate VA morphology using different imaging modalities. For reference, micro-CT was applied and compared to two different clinically available imaging modalities; micro flat panel volume computed tomography (mfpVCT) and multi-slice computed tomography (CT). From these data a clinically suitable model for grouping the VA morphology according to a newly developed “vestibular aqueduct score” (VAS) was developed by application of a virtual 3D model based on the angle relative to the semicircular canals (SCC). Furthermore, it was evaluated, as a first test of the VAS whether the score might be used to group the outcome of patients who were treated by saccotomy, with respect to their success in reduction of symptoms.

## Methods

### Temporal Bone Specimens

Temporal bone specimens (TBS) were used for the development of this specialized methodology. Ten TBS were scanned in an experimental micro-CT with a slice thickness of 18 μm, which served as a reference. In addition, 37 specimens were examined by CT with a slice thickness of 600 μm, plus an additional mfpVCT scan with 197 μm slices. There were 35 specimens from the right side, and two from the left side.

### Patient Selection

For control measurements, 42 CT temporal bone scans were used from selected patients with normal hearing and vestibular function. All patients received radiological imaging prior to middle cranial fossa operations for vestibular neurinoma surgery of the contralateral ear. In addition, 52 patients, who had a confirmed MD diagnosis according to the AAO-HNS classification (24), Gibson Score (25) or positive Ecohgh results for MD, were selected for the study group. All 52 patients were treated by saccotomy (26) and were examined by CT and cranial magnet resonance imaging for exclusion of a retrocochlear lesion. Anamnestic data were used and stored anonymously. The study was conducted in concordance with local guidelines and principles of the Declaration of Helsinki and Good Clinical Practice and was approved by the local ethics committee at the University of Würzburg (20191127/02).

### Imaging

Micro-CT measurements were performed using a MetRIC setup with the following parameters: Tube voltage = 120 kV; power = 4 W; exposure time = 200 ms; 15 averaged images; 2 mm aluminum and 0.38 mm silicon filters; slice thickness = 18 μm (27). CT scans were recorded by the multi-slice scanner SOMATOM Definition AS+ (Siemens Healthcare AG) with the following parameters: Tube voltage = 120 kV; tube current = 38 mA; pitch = 0.55; collimation = 0.6 mm; slice thickness = 600 μm. mfpVCT scans were performed on an Axiom Artis (Siemens Healthcare AG) using the following parameters: 20s DynaCT head protocol; tube voltage = 109 kV; tube current = 42 mA; pulse length = 3.5 ms; rotation angle = 200°; frame angle step = 0.4°/frame; slice thickness = 197 μm.

### Imaging Processing

**Figure 1:**
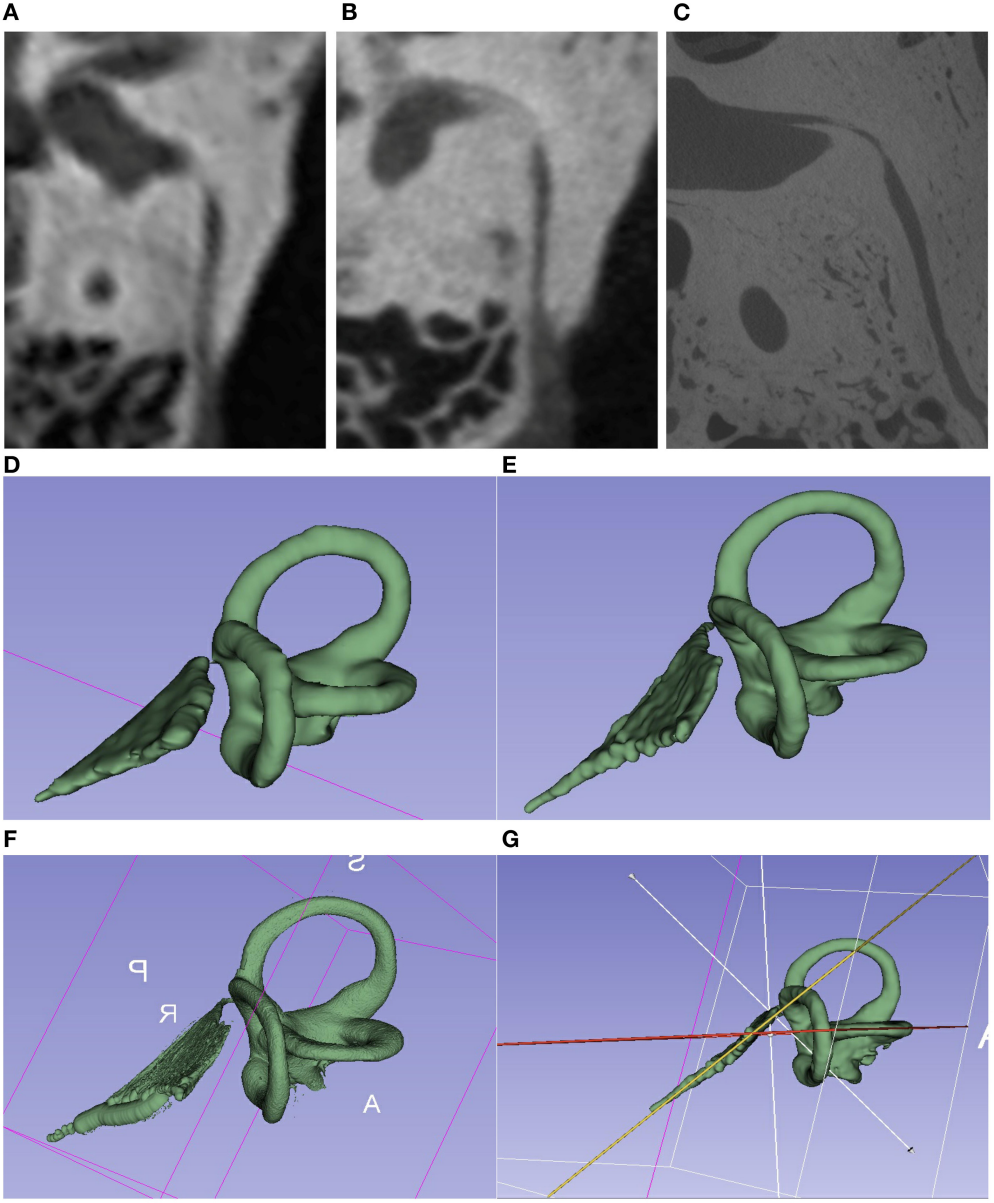
Comparison of the imaging techniques of the same TBS. **(A–C)** Images of different image quality [**(A)** = CT 600 μm; **(B)** = mfpVCT 197 μm 600 μm; **(C)** = micro-CT 18 μm]. VA seen in its full length in the axial plane. **(E–G)** 3D model created with the 3D slicer software using the segment editor tool. [**(D)** = CT; **(E)** = mfpVCT; **(F)** = micro-CT]. **(G)** Presentation of the angle measurement technique.

The images were saved as DICOM files and imported into the Horos® Medical Viewer (version 3.3.5) and 3D Slicer® (version 4.10.2) for processing. The 3D Curved MPR (multiplanar reconstruction) function in Horos® was used to determine the length of the VA from the entrance at the vestibule up to the exit at the ES. The axial plane was always selected as the display plane (Figure 1). Each point was placed in the middle of the lumen.

The freely available software 3D Slicer® was used to create a 3D model of the inner ear. As described in the work of Dhanasingh, Dietz (28), the Segment Editor Tool was used as the basis for creating a 3D model of the SCC and the VA. First, the region of the SCC and the VA was selected *via* the function “Crop Volume” and “Create New Annotation ROI” and cropped to this region with the settings Isotropic Spacing, Spacing Scale 0.5, Interpolator Linear and Interpolated Cropping. In the Segment Editor, the threshold function can then be used to adjust the gray value so that the SCC and VA are marked as accurately as possible. The marked threshold range was saved as a mask (“Use for masking”) and manually traced. This process was always done in the axial plane first and then in the other planes. With the existing 3D model (Figure 1), the extension “Angle Planes” made it possible to define 2 planes, and to measure an angle between these planes using the Python Interactor (Text Code Angle 3D Slicer) (29). With respect to the planes, both the cutting plane of the VA and one of the SCC were used, and then the angle in between was then measured (see Supplementary Video 1).

### Saccotomy

The standard procedure of saccotomy includes an antrotomy followed by a mastoidectomy with thinning out the bone over the dura of the posterior fossa and exposing the sigmoid sinus. For identification of the ES, the lateral SCC is thinned out until the endost and the so called “blue line” is visible. Then the posterior semicircular canal and its endost are identified. Afterwards the posterior SCC is hypothetically bisected by a line, drawn in the same direction as the blue line of the lateral SCC. The ES can be than be identified dorsally to the caudal half of the posterior SCC and medially to sigmoid sinus as a duplicate of the dura. Finally, the ES is slit to open, and a silicone triangle inserted, which results in permanent drainage of perilymph from the ES into the mastoid.

### Statistical Analysis

Descriptive data analysis (mean value, 95% confidence interval (CI) and standard deviation) was performed with Microsoft Excel. The paired *T*-test and the two-sample *T*-test were employed to test for statistical significance. Null hypothesis was rejected if *p* was determined smaller than 0.05. In addition, the intraclass correlation (ICC) and Cronbach alpha were evaluated using SPSS (IBM). According to Koo and Li (30), a correlation of >0.9 for the ICC was considered to be adequate. As a test model, two-fold mixed with absolute agreement and a confidence interval of 95% was used. For the Cronbach Alpha, values of α > 0.9 were considered to be highly reliable. When three or more groups were compared the one-way ANOVA was used. The 3D diagrams were created with Excel using a template (31), and for other diagrams Graphpad Prism 8.3® was used.

## Results

### Comparison of Angle and Length of the VA Between Micro-CT and mfpVCT in TBS

To establish the method, angle measurements of the VA in relation to all the SCC were performed in 10 TBS scanned by micro-CT images (18 μm). In addition, mfpVCT (197 μm) was performed which is the imaging modality with the highest possible resolution in humans. Data are presented in Table 1.

**Table 1:** Anamnestic data of saccotomy patients before and after surgery.

	**Preoperatively**		**Postoperatively > 6 weeks**	**Postoperatively <6 weeks**
Vertigo	100%	Ø	38%	28%
		<	32%	36%
		=	30%	36%
Tinnitus	98%	Ø	71%	60%
		=	29%	40%
Aural fullness	82%	Ø	77%	68%
		=	23%	32%

*After surgery is divided into less than and greater than 6 weeks. “Ø” = no symptoms; “ < ” = less symptoms than before surgery; “=” = symptoms like or worse than before surgery*.

All measurements were performed twice to check reliability. Evaluation of the T-test with dependent samples of the 1st and 2nd test series from both recordings consistently showed no statistically significant differences (micro-CT: *p* = 0.46, ICC = 0.99, α = 0.99; mfpVCT: *p* = 0.15, ICC = 0.99, α = 0.99) and a difference of 0.3° for micro-CT and mfpVCT, so the null hypothesis was accepted.

**Figure 2:**
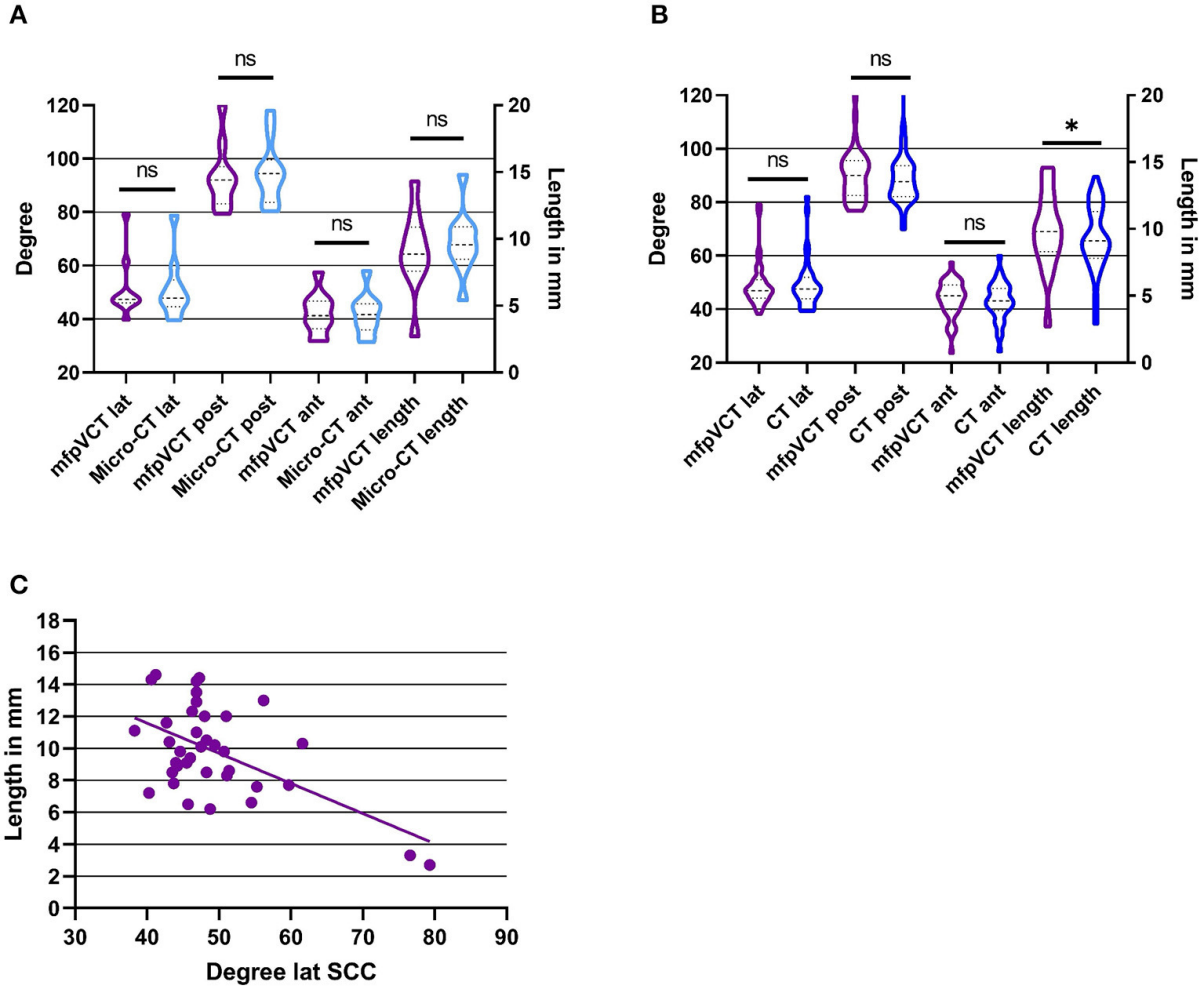
Results of angle and length measurements of the TB specimen in comparison with the different image techniques. **(A)** mfpVCT and micro-CT (*n* = 10), **(B)** mfpVCT and CT (*n* = 37), and **(C)** Correlation of length and angle of the lateral SCC and the VA. *p < 0.05.

When comparing the results of angle measurements of the micro-CT and mfpVCT images, there were no significant differences (lat SCC: difference = 0.07°, *p* = 0.94; post SCC: difference = 1.7°, *p* = 0.11; ant SCC: difference = 0.3°, *p* = 0.43). Thus, the mfpVCT images can be used as a reference. The intraclass correlation and the Cronbach alpha were also evaluated and showed a correlation coefficient of greater than 0.9 (ICC = 0.98–0.99) and α > 0.9 (α = 0.97–0.99) when comparing the series of measurements and comparing micro-CT to mfpVCT, which complies with a very reliable result (Figure 2A).

The comparison of the VA length determination of micro-CT and mfpVCT showed no significant differences (difference = 0.5 mm, *p* = 0.1, ICC = 0.93, α = 0.92). The values of the micro-CT were slightly longer than those of the mfpVCT since the exact entry into the vestibule is shown only in the micro-CT (Figure 1D). Nevertheless, the mfpVCT is sufficiently accurate (Figure 2A).

### Comparison of Angle and Length Between mfpVCT and CT in TBS

Normally, only lower-resolution images of patients are clinically available, so it was necessary to check whether the results of the high-resolution TBS images were consistent even with thicker layers. To investigate this, 37 TBS (including the 10 TBS scanned by micro-CT) were scanned by mfpVCT and CT. All data are presented in Table 1.

The angle measurements were performed twice with the result that both series were nearly identical (CT: difference = 0.3°, *p* = 0.2, ICC = 0.98, α = 0.99). The measurements of the angle in CT showed no significant differences compared to mfpVCT (lat SCC: difference = 0.1°, *p* = 0.87; post SCC: difference = 1.1°, *p* = 0.15; ant SCC: difference = 0.8°, *p* = 0.2; α = 0.94–0.97, ICC = 0.94–0.97). The comparison of the length determination of mfpVCT and CT showed significantly shorter values in CT (difference = 0.5 mm, *p* = 0.03, ICC = 0.88, α = 0.87) (Figure 2B).

A correlation analysis was performed between the length of the VA and the respective angle. Only for the lateral SCC there was a clear tendency (r^2^ = 0.32, *p* = 0.0003), showing that with a higher angle, the VAVA were relatively smaller. For the other SCC, there was no correlation (r^2^ = 0.17, *p* = 0.03) (Figure 2C).

### Angle and Length Measurements in Patients

Measurements in patients were performed in CT scans. The VA length in non-MD patients was 9.3 mm (range: 5–16.1 mm, SD: 2.4 mm, 95% CI: 8.8–10.4 mm), and the angle to the lateral SCC was 55° (range: 35.6–92.6° mm, SD: 13°, 95% CI: 52.5–60.8°), the posterior 93.9° (range: 79.6–147.1°, SD: 14°, 95% CI: 92.6–102.1°) and the anterior 43.7° (range: 30.3–59.2°, SD: 7°, 95% CI: 41–45.6°).

The VA length in MD patients was 7.9 mm (range: 3.6–13.0 mm, SD: 2.4 mm, 95% CI: 7.3–8.6 mm). The angle to the lateral SCC was 71.4° (range: 29.5°–125°, SD: 19.4°, CI: 67.1°–77.5°), the posterior 102.7° (range: 57.5°–157.8°, SD: 20.8°, CI: 97.9°–109.6°), and the anterior 37.8° (range: 4.4°–75°, SD: 15.2°, CI: 33.2°–41.7°).

**Figure 3:**
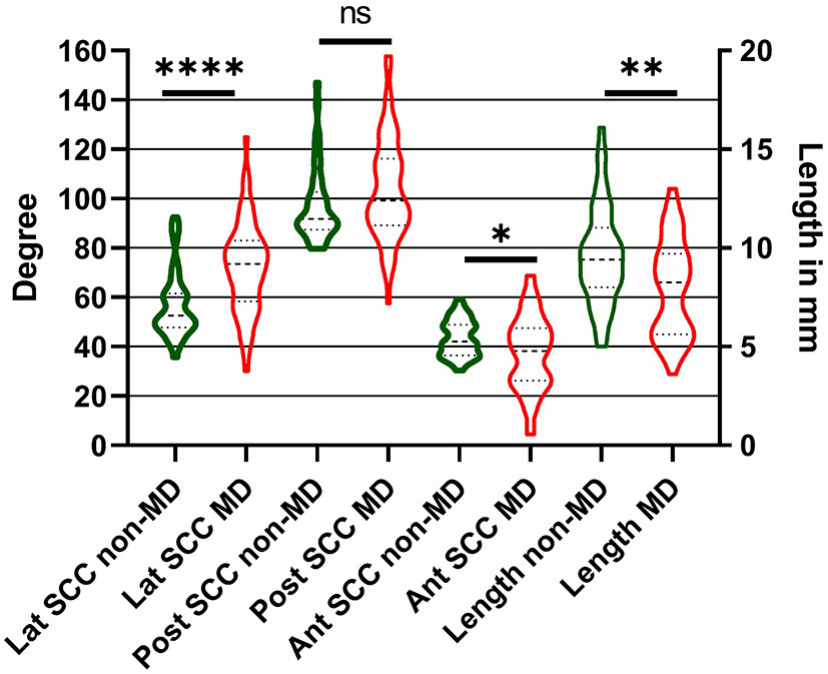
Results of angle and length measurements of MD and non-MD patients in comparison. Width of the bars show the distribution. Differences between the measurements were significant for the lateral and anterior SCC and the length between MD and non-MD patients. *p < 0.05; **p < 0.01; ****p < 0.0001.

In comparison with the non-MD patients, there is a significant difference between the results of the lateral SCC (difference: 16.4°, *p* = 0.0001), the anterior SCC (difference: 5.9°, *p* = 0.03) and length (difference: 1.4 mm, *p* = 0.03). For the posterior SCC (difference: 8.8°, *p* = 0.1) no significant difference was found (Figure 3).

### Development of the VAS

**Figure 4:**
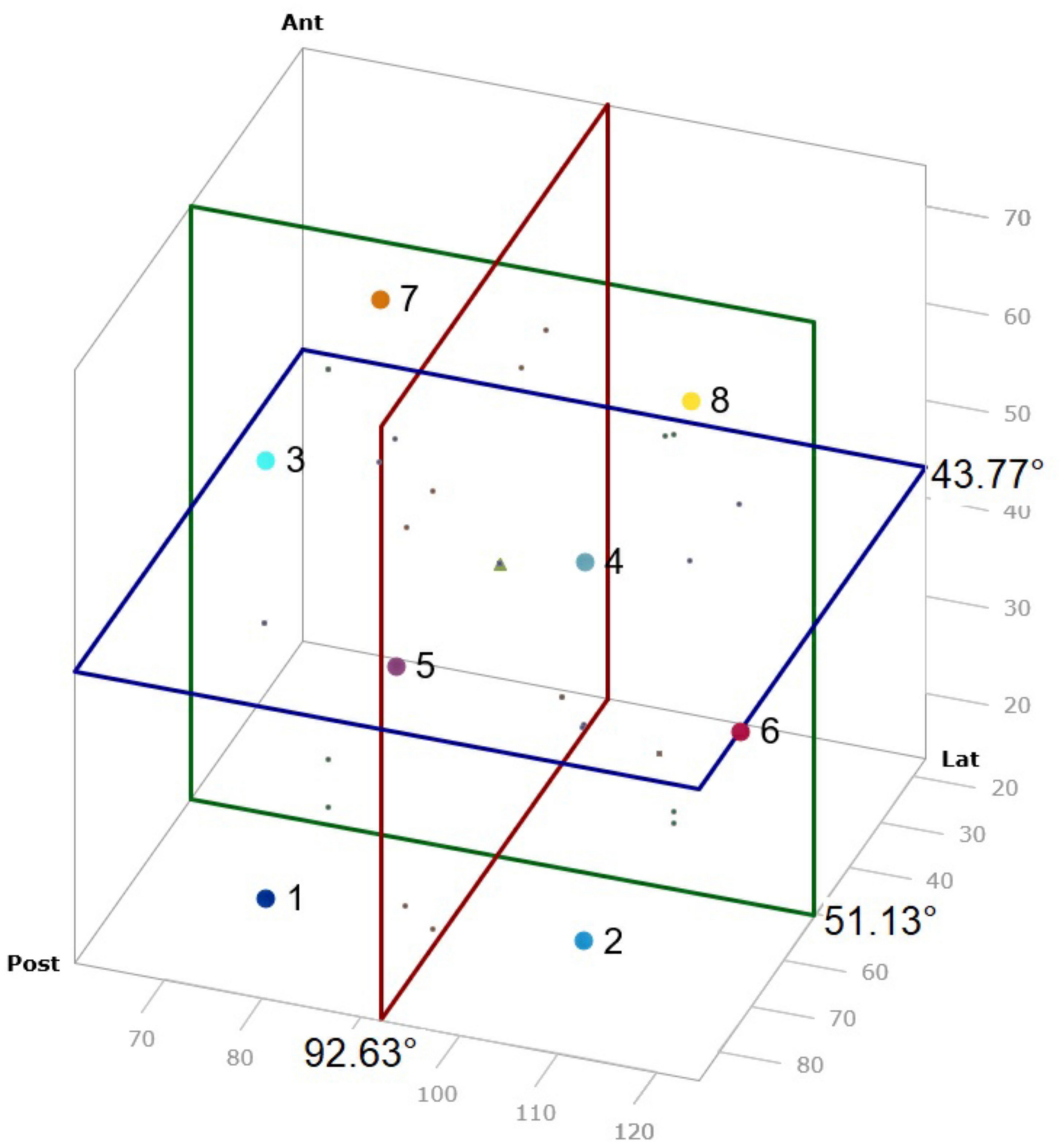
VA score scheme as 3D diagram. Points mark the different VAS groups.

To facilitate the comparison of the angle measurement results of the VA, a score was developed using the mean value and twice the standard deviation of the angle measurements to form a 3D diagram, and then dividing this into 8 cubes which represents the various VAS groups. Therefore, all SCC angle results could be represented in one value and results were categorized. Each axis of the 3D diagram was divided into lower and higher than the mean value to obtain 8 groups (Figure 4). The averaged mean value of all non-MD VA and TBS was used, which included 79 datasets. These were 51.13° ± 20.4° (2x SD) (range: 35.6–92.6°, SD: 10.2°, 95% CI: 49.7–54.3°) (lateral SCC), 92.63° ± 24.6° (2x SD) (range: 75–147.1°, SD: 12.3°, 95% CI: 89.6–95.1°) (posterior SCC) and 43.77° ±14° (2x SD) (range: 23.6–59.2°, SD: 7°, 95% CI: 42.1–45.3°) (anterior SCC).

### Assignment of the VA to the VAS

**Figure 5:**
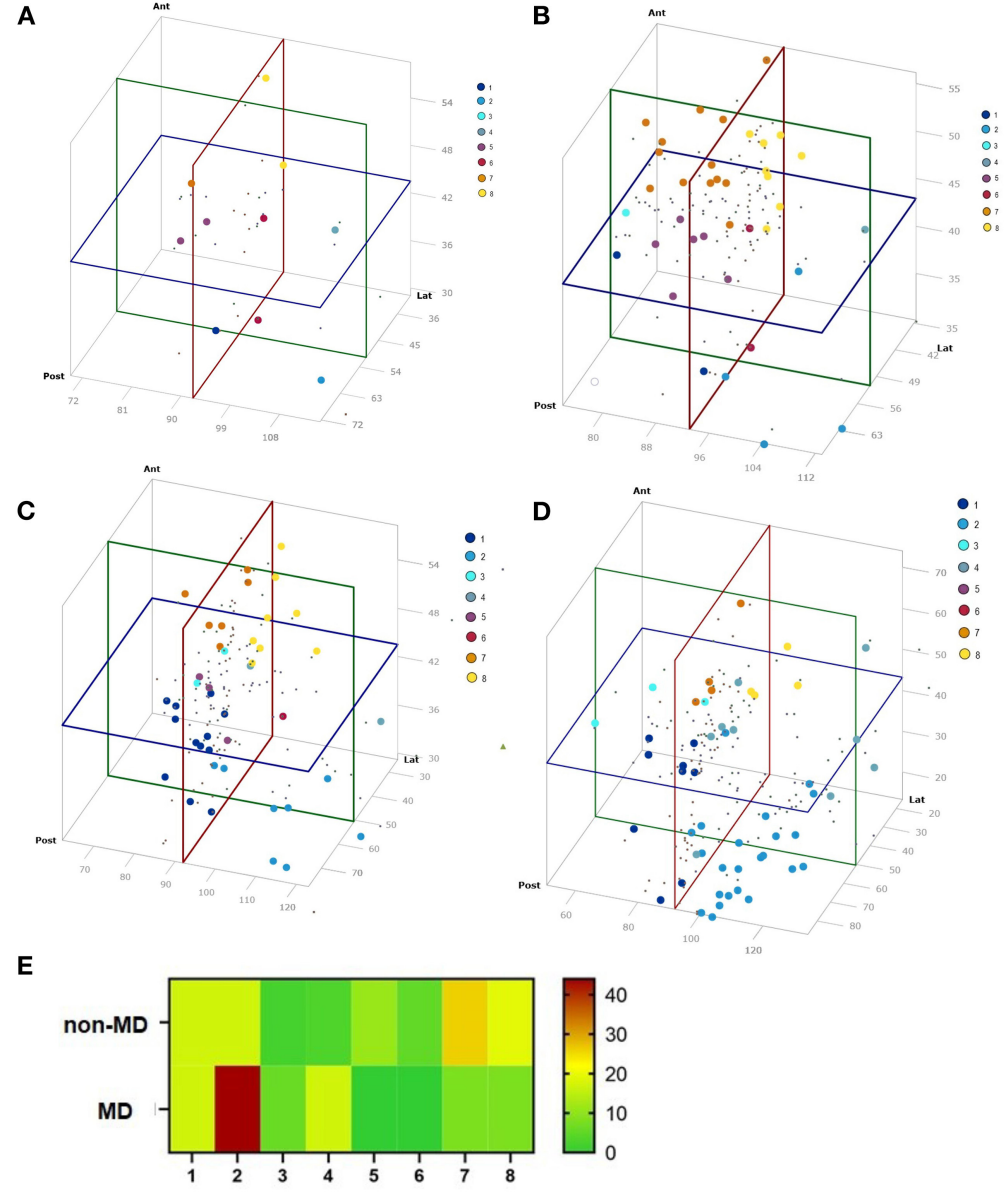
Results of the angle measurements visualized in a 3D diagram. Colors represent the VAS group. **(A)** TBS micro-CT (*n* = 10), **(B)** TBS mfpVCT (*n* = 37), **(C)** non-MD patients (*n* = 42), **(D)** MD patients (*n* = 52), and **(E)** Distribution of each group according to the VAS. MD patients are concentrated in group 2, as compared to non-MD patients and TBS, where the distribution is more balanced.

The VA of the 10 TBS, measured by micro-CT, were assigned to the respective groups 1–8 of the VAS: 1 (10%), 2 (10%), 4 (10%), 5 (20%), 6 (20%), 7 (10%), 8 (20%) (Figure 5A). The VA of the 37 TBS, measured by mfpVCT, could be matched to the respective groups 1 (5%), 2 (16%), 3 (2%), 5 (14%), 6 (5%), 7 (32%) 8 (26%) (Figure 5B). The non-MD VA could be matched to the VAS: 1 (29%), 2 (19%), 3 (5%), 4 (5%), 5 (7%), 6 (2%), 7 (14%), 8 (19%) (Figure 5C) and the VA of MD patients to the VAS: 1 (17%), 2 (44%), 3 (6%), 4 (17%), 7 (8%), 8 (8%) (Figure 5D). Analysis by heat map displayed an even distribution in TBS and non-MD patients, whereas VAS of MD patients grouped mainly in VAS 2.

### Anamnestic Data MD Patients

The medical history of each patient was evaluated both preoperatively and postoperatively according to the scheme of the Gibson score and AAO-HNS criteria. Patients received an EcochG for diagnosis. This showed definite EH in 81.6%, marginal evidence in 12.2%, and no evidence of EH was found in 6.2% of the cases. The hearing level was also examined. The pure tone audiogram was evaluated at 500, 1,000, 2,000 and 4,000 Hz at initial evaluation, just before and after surgery. PTA^4^ hearing threshold (AC) of 46 dB HL was calculated at initial presentation and a PTA^4^ bone conduction threshold of 44 dB HL. This deteriorated until before surgery (time period 126 weeks) to 67 dB HL (AC) and 60 dB HL BC. Analysis of the data with ANOVA, showed a significant difference in the progression of hearing loss from initial hearing test to after surgery (*p* = 0.01).

### Correlation VAS and Outcome Saccotomy

To apply the VAS in a clinical setting, MD patients who were operated by saccotomy were chosen. In every operation, it was possible to expose the ES. Results of the saccotomy were categorized as successful and not successful. The success criteria were defined as follows; patients who reported being completely free of vertigo or just mild persistent vertigo were considered successful. Patients who continued to suffer from dizziness (same or worse than before surgery) were considered unsuccessful.

Post-surgical evaluation timeframes were divided in to up to 6 weeks and more than 6 weeks after surgery. The success rate of the saccotomy in the observation period <6 weeks was 72% and more than 6 weeks, 64% (Table 1). In 8 patients, it was stated that the petrous bone was very compact, 11 patients had a fibrous sac.

**Figure 6:**
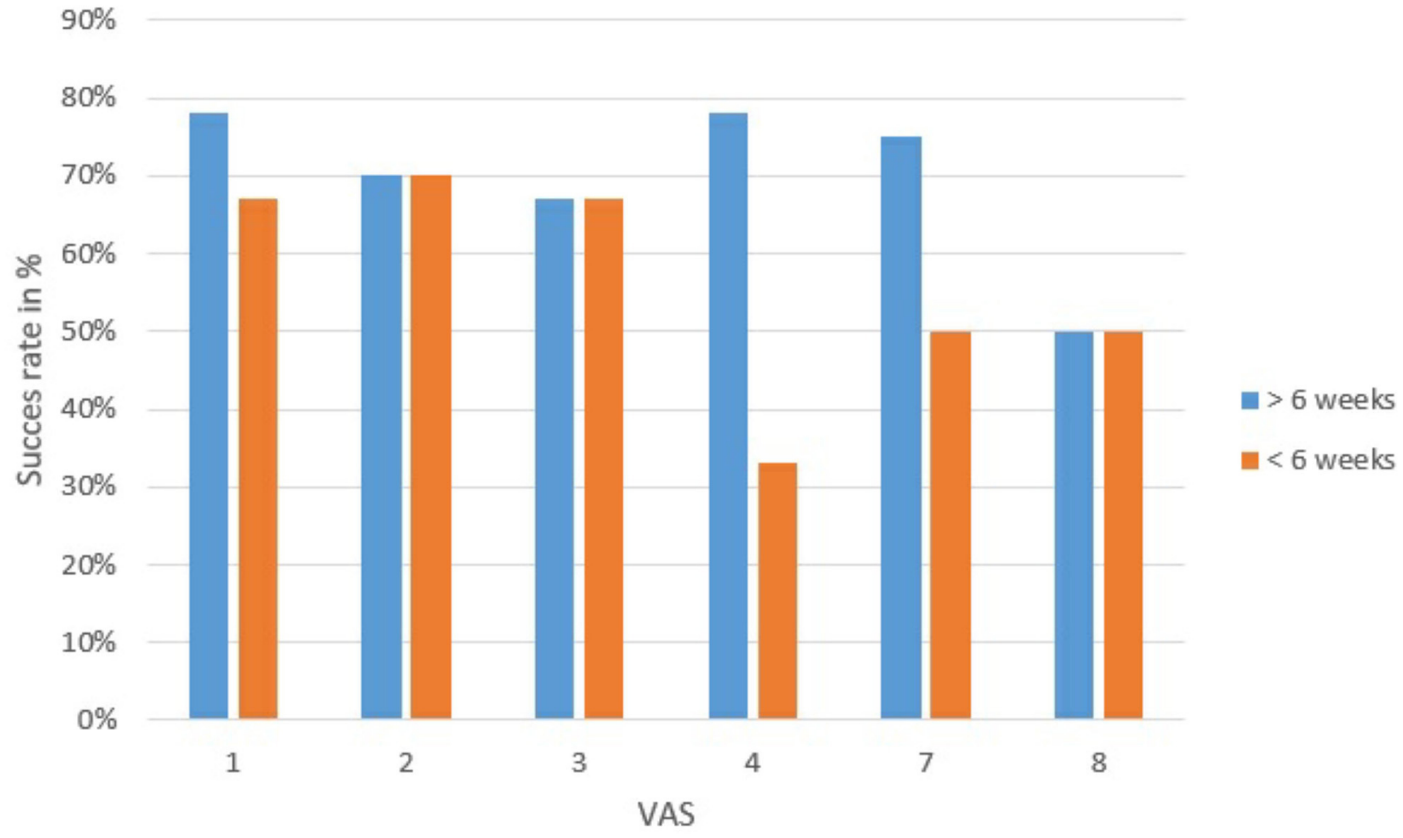
Outcome of the saccotomy compared to each VAS divided into 6 weeks before and after surgery. Successful outcome means that patients have not vertigo or better tan before operation. VAS 4 had the most patients with unsuccessful treatment.

To correlate the outcome of the saccotomy with the VAS, the success rate of each VAS group was calculated. The lowest success rate in the observation period <6 weeks was in VAS 8 with 50% and 2 with 70% (Figure 6A). After the period of 6 weeks, VAS 4 showed a success rate of only 33%. The other groups did not differ much (Figure 6B).

## Discussion

In the present study, it was shown that it is possible to measure the angle of the VA relative to the three SCC in CT scans as a feasible and comparable method to other imaging modalities with higher resolution. The values were significantly different in MD patients compared to non-MD patients, which resulted in a different distribution of the VA according to a newly developed VAS score (Figure 5E). These results, and especially the improved method of grouping the VA with respect to their position to the SCC in the VAS offers a novel approach for more differentiated diagnostics of MD. It might also be of further clinical relevance since the newest theory of the cause of an MD attack is based on anatomical changes of the endolymphatic duct, in particular a reduction of the lumen, which results in overfilling of the endolymphatic sinus and a potential discharge of endolymph through the valve of Blast into to utricle (5). Consequently, the morphological changes might be more prominent in specific groups of the VAS, resulting in a higher rate of MD or an altered susceptibility to treatment options.

Many studies have already attempted to more accurately identify the pathophysiology of MD. Bachinger et al. (20) showed that the VA in MD patients can have a different position, and in other studies (22, 32, 33) it was recently shown that narrow bony VA favors EH, and thus the morphology of the VA plays a role in the expression of MD. These works were facilitated by new and improved imaging technologies such as high-resolution CT data.

A possible limitation of all radiologically based studies in the field of MD is the slice thickness of the images. Particularly when the slice thickness increases, the VA, with its very narrow lumen, is often not easily or no longer recognizable (Figure 1). In the study by Stahle and Wilbrand (19), only 65% of the VAs of the ears affected by MD were visible, whereas 81% of the VAs of the ear not affected by MD were clearly detectable. They stated that a better imaging technique is necessary to perform a reliable delineation of VAs. To further address this problem, different imaging modalities were investigated. It was shown that the angle of the VA to the SCCs can be detected without significant differences in CT, but the length differed significantly between mfpVCT and MSCT because of the reduced resolution. For this reason, the length of the VA was not used for the development of the VAS score.

To our knowledge, the measurement of the angles of VA to the 3 SCCs has not yet been published before. All SCCs were used as markers because they are at a constant angle to the axes of the body. As early as 1974, Wilbrand measured the angle between anterior SCC and VA (34). Thirty-five unselected temporal bones were examined, in which an average angle of 45° between the VA and anterior SCC was found. This corresponds very well with the results presented here (Table 1) and is a further confirmation that VA angle measurements provide reliable results. Another observation reported by his group was that longer aqueducts showed a smaller angle between the entrance and exit path of the VA compared to shorter aqueducts. The results showed that there was also a tendency toward a correspondence between angle size and length. Similar to the present study, a correlation between the angles of the lateral SCC and the length of the VA could be determined (Figure 2C), which shows that these two values are probably the two most important for identification of different anatomical variations of the VA.

Based on the results from the TBS, it was revealed that CT images with a higher slice thickness (600 μm), which are normally performed in the clinical setting, can be used to perform angle measurements and to create a 3D model. Both the results of the mfpVCT and the MSCT corresponded to the reference of the micro-CT.

For the 3D model, the mean plus two SD of angle measurements performed in non-MD patients and TB specimens (*n* = 79) was chosen since they represent VA not affected by MD. It cannot be ruled out that an MD patient could have potentially been included in the anonymously used TBS, but none of the angles were different from the group of non-MD patients, which represent a group of the contralateral side of vestibular schwannoma patients not suffering from MD. Consequently, the angle measurements of the 79 VA can thus be used as a control. The mean plus two SD was chosen since in normally distributed data, which the angle measurements are, this range represents 94.45% of the data. Following these statistical considerations, the VAS should be able to group the angle values of the VA in relation to the SCC in an appropriate mode. Indeed, this was shown by the fact that the VAS scores of the TBS and non-MD patients had a broad and even distribution, whereas 90% of MD patients were assigned to the groups 1, 2 and 8 (Figure 5E).

The results of the angle measurements showed significant differences between non-MD and MD VA with higher angle values to the lateral and posterior SCC and lower values to the anterior SCC in MD VA. Moreover, the standard deviation was higher in MD patients, because of a much larger range of the results.

Interestingly, there were 10 aqueducts within the MD group which were particularly noticeable with a very short length (<5.5 mm), and a flat angle to the lateral (<74°) and posterior SCC (<110°). They were assigned to group 2 and 4 of the VA score. They resemble the “hypoplastic” type described by Bachinger et al. (20). In their study, they divided the VA into hypoplastic and degenerative with respect to their angle between the entrance and exit of the VA. This allowed the two different types of disease pathologies to be distinguished. In this method of classification, only the group of hypoplastic VA can be clearly assigned to MD, since the form of the degenerative type corresponds to that of normal adults.

The present investigation was performed using a new measurement technique known as the “3D Curved MPR” (35). It is the first time that the VA length has been analyzed using this technique, but the results are (Table 1) similar to those reported in the literature (18, 19).

One obstacle to the presented method is that it still cannot be used in everyday clinical practice. As the 3D Slicer software is at present only suitable for scientific purposes, this method cannot yet be used as a clinical diagnostic tool in its current form since calculations must still be done manually. Therefore, a system will be required which automatically marks the SCC and VA and can display them in 3D, thus facilitating use in clinical practice.

MD patients treated with saccotomy were included because these patients had a full set of diagnosis prior to surgery. To include as much data as possible for this work, all existing anamnestic parameters were noted and based on these data, the Gibson score and AAO-HNS was calculated. However, problems with these diagnostic guidelines are the interpretation of the scores and their dependence on patient‘s information, since not all parameters can be measured clinically. This is why an additional score with radiological features might help to distinguish patients more clearly and help with the diagnosis of MD, as the Hydrops MRI, which has been shown to be applicable (36).

By using the data of the success of saccotomy a first test was performed for the application of the VAS. As shown in Figure 6, patients in VAS score 4 are the group of patients with the lowest success rate (33%). In contrary, patients grouped patients grouped to VAS score 1 to 3 are those with the best success rates. However, it has to be mentioned that due to uncertainty surrounding whether saccotomy is a valid treatment for MD (37), and the relative small number of patients, these data have to be interpreted with caution. In the future, further studies correlating the VAS with other findings, such as effectiveness of different therapies, but also with the variant symptoms of MD, should be performed on larger series of patients to determine whether the VAS is a valid tool to stage MD patients.

## Conclusion

By the application of different imaging modalities, it could be shown that angle measurements of the VA are reliable and can be used to enhance the diagnosis of patients with MD. Length and angle results from these patients differed from non-MD patients. From the angle measurements of the temporal bone specimen and the non-MD patients, a 3D model was constructed, and the VAS score developed. This score offers a new method for grouping MD patients with respect to the morphology of their VA, making it possible to group MD patients, for example, with regard to the success of a specific MD treatment.

## Data Availability Statement

The raw data supporting the conclusions of this article will be made available by the authors, without undue reservation.

## Ethics Statement

The studies involving human participants were reviewed and approved by Ethic Comittee of University Wuerzburg Institute for Pharmacology and Toxicology Versbacherstraße 9, 97078 Wuerzburg, Germany. Written informed consent for participation was not required for this study in accordance with the national legislation and the institutional requirements.

## Author Contributions

LN developed, executed and analyzed the measurements, and wrote the manuscript. LI helped with statistical analysis, x-rays, and writing the manuscript. MB, WS-D, JT, and RH helped developing the method and contributed anamnestic data. TN, SZ, and DA performed the x-rays. KR helped creating the method, performing measurements and analysis, and wrote the manuscript. All authors contributed to manuscript revision, read, and approved the submitted version.

## Funding

This publication was supported by the Open Access Publication Fund of the University of Wuerzburg.

## Conflict of Interest

The authors declare that the research was conducted in the absence of any commercial or financial relationships that could be construed as a potential conflict of interest.

## Publisher's Note

All claims expressed in this article are solely those of the authors and do not necessarily represent those of their affiliated organizations, or those of the publisher, the editors and the reviewers. Any product that may be evaluated in this article, or claim that may be made by its manufacturer, is not guaranteed or endorsed by the publisher.
